# Pandemic Racism: Lessons on the Nature, Structures, and Trajectories of Racism During COVID-19

**DOI:** 10.1007/s11673-023-10312-0

**Published:** 2023-11-02

**Authors:** A. Elias, J. Ben

**Affiliations:** Alfred Deakin Institute for Citizenship and Globalisation, Deakin University, 221 Burwood HWY, Burwood, Victoria 3125 Australia

**Keywords:** COVID-19, Pandemic-racism, Discrimination, Structural racism, Racial disparities, Anti-Asian racism

## Abstract

The COVID-19 pandemic has been one of the most acute global crises in recent history, which profoundly impacted the world across many dimensions. During this period, racism manifested in ways specifically related to the pandemic, including xenophobic sentiments, racial attacks, discriminatory policies, and disparate outcomes across racial/ethnic groups. This paper examines some of the pressing questions about pandemic racism and inequity. We review what research has revealed about the nature and manifestations of racism, the entrenchment of structural racism, and trajectories of racism during COVID-19.

## Introduction

In early 2020, the world started facing one of the most acute health crises in recent history, with significant social, economic, ethical, and political repercussions. Racism, a system of oppression that has been unfolding globally for centuries, had unique COVID-19 manifestations from day one. It drove physical and verbal attacks against Asian people and other minorities, and ubiquitous calls for foreigners to “go home”; circulated in political discourses and pushed on new online platforms; manifested in discriminatory policy responses and restrictions, disproportionately targeting migrants as “suspect” spreaders; and could be gauged through disparate rates of pandemic-related infections, hospitalizations, and deaths, alongside other critical health and socio-economic outcomes (Dhanani and Franz 2020; Shi, et al. 2022).^1^

More than three years on, the world is struggling to achieve a “post-pandemic” recovery. And while the pandemic has largely subsided (WHO 2023),^2^ its long-term consequences and legacies continue to affect us. It is now an appropriate moment to consider what we have learned about racism during COVID-19, drawing on recent theoretical and empirical insights garnered from this period. This paper examines some of the pressing questions about racism and inequity during the pandemic. We discuss what the pandemic has revealed about the nature and manifestations of racism, the entrenchment of structural racism, and trajectories of racism during the pandemic and beyond. We discuss lessons learned in relation to post-pandemic recovery, with an eye to countering racism and handling future crises.

## The Nature of Racism

During the outbreak of COVID-19, racism has taken pandemic-related forms, feeding on climates of exclusionary nationalism and xenophobia (Elias, et al. 2021), while some scholars have characterized racism itself as another pandemic (Godlee, 2020) or a “syndemic,” concurrently rising with COVID-19 (Gravlee, 2020; Njoroge, et al. 2022). An inherently global event, the pandemic broke out simultaneously with the emergence and advancement of new technologies that helped streamline, share, and spread racism on a worldwide scale. Owing to the ubiquity of new media and digital technologies, COVID-racism has been remarkable in the speed and extent of its global spread (Depoux et al., 2020; Dionne and Turkmen 2020). Specifically, media had an important role in encouraging scapegoating and Othering (Ristic and Marinkovic 2022), circulating anti-Chinese and anti-immigrant discourses.

COVID-19 was a watershed moment for online racism (e.g., Croucher, et al. 2020). It generated a surge in online activity and novel challenges to regulating misinformation, hate, and discriminatory content, as illustrated by the rapid evolution of Sinophobia on Web communities (Schild, et al. 2020). The sharing of racism globally was further enabled by widespread biopolitical measures to control the virus, such as geofencing, social distancing, and lockdowns, extended to blocking out and controlling select minorities (Guma, et al. 2023; Sylvia 2020).^3^

Notwithstanding these broad currents, racism manifests everywhere differently, locally (Forrest and Dunn 2006) and transnationally (Kobayashi and Peake 2000). The pandemic confirmed observations about the malleability of racism. It provided new contexts to old ideas about danger and contagion, immorality, and crime. These contexts were remoulded into well-established historical frames of prejudice (Bavel, et al. 2020), echoing, for example, historical naming practices (e.g., “Chinese virus” following other links between pandemics and nations/peoples), longstanding fears about Asian migrants, or deep-seated anxieties about minorities as criminalized. “Race” too has been continuously reinscribed in processes of Othering and (re-)linked with ideas of health and morality, with racial difference as existing biologically via contagious bodies, and culturally through (im)moral practices. One worrisome development during the pandemic was the policing and surveillance that resulted in outbreak localization and lockdown measures disproportionately affecting marginalized communities. There were various cases where racialized and migrant neighbourhoods were unfairly targeted, leading to their criminalization (Hendl, et al 2020).

Additionally, the pandemic was a litmus test of the state and transformations to blatant forms of racism. In recent decades, covert forms of racism have become increasingly prevalent (Seet and Paradies 2018). Reduction in openly racist expressions has been (erroneously) taken as evidence that racial injustice has been overcome and that we have entered a “post-racial” era (see a critique in Lentin 2021). The pandemic dealt a serious blow to such myths, lifting the thin veil apparently covering blatant racism. Openly racist expressions quickly came into view, with explicitly discriminatory language widely hurled from heads of state to users of online dating apps (Li and Chen 2021). The torrent of anti-Asian attacks early into the pandemic showed that capacities for violence, abuse, and hatred remain close to the surface, ready to rise under certain conditions (e.g., xenophobia, uncertainty, and inciting authorities) (Ben and Elias, under review). Subtler forms of racism were still expressed during the pandemic, while some of its hidden structural forms have been convincingly exposed.

Going forward, these characteristics of COVID-racism call for greater accountability from politicians and media in handling future pandemics, and for closer regulation of media through which racism circulates. Likewise, in pre-empting the next pandemic, intersections between xenophobia, scapegoating practices, and abuse of online and biopolitical technologies, and their links with misinformation indicate a need for a transparent and timely dissemination of public health information.

## Structures and Disparities

While COVID-19 has become a context for outbursts of racism and xenophobia, it also revealed and amplified existing structural inequalities and racial disparities (Tai, et al. 2021). Health disparities have many causes, including individual/collective behaviours, biological factors, economic conditions, educational opportunities, and healthcare access. Racism and discrimination are crucial determinants of health, and as such have contributed to the disparities associated with the pandemic (Webb Hooper, et al. 2020).

Across countries, several studies have indicated that people from minority racial and ethnic backgrounds were more likely to be infected, to experience severe complications, and to die because of COVID-19 (Webb Hooper, et al. 2020). In the United States, African American and Hispanic populations have been among the most affected (Magesh, et al. 2021; Yancy 2020) while Asian Americans had the highest risk of admission in intensive care unit (Magesh, et al. 2021). Several chronic conditions that are associated with the disparity in adverse COVID-19 outcomes, and hypertension, obesity, diabetes, and asthma, were more prevalent among African Americans compared with white people (Wang, et al. 2021; White, et al. 2021). Studies indicate that African Americans and other minorities were disproportionately impacted by COVID-19-related mortality (Bassett, et al. 2020; Bhala, et al. 2020; Mackey, et al. 2021; Zanolli 2020). Associations were also found between interpersonal/structural racism, negative COVID-19-related experiences and greater risk for postpartum depression and anxiety in a longitudinal study of Black birthing cohort (Njoroge, et al. 2022).

In the United Kingdom, studies suggest that minority groups may experience higher COVID-19 incidence and severity due to possible pathophysiological differences in susceptibility or response to infection (which may be risk factors for COVID-19 severity), as well as genetic predispositions (Khunti, et al. 2020). Several studies have found people from ethnic minority backgrounds were more widely and acutely affected than other people, although rates seem to vary across studies (Bhala, et al. 2020; Patel, et al. 2022), and between minority groups (Platt and Warwick 2020).

Data about COVID-19 infections, hospitalization, and death across ethnic groups in other countries have been very limited and consisted at the early phase of several studies that estimate the severity of illness and death based on existing risk factors. In New Zealand, for example, Maori people were substantially more likely to have comorbidities associated with higher risk of severe COVID-19 illness and death (McLeod, et al. 2020; Steyn, et al. 2020; Whitehead, et al. 2023). In Australia, researchers working with Indigenous people have asserted that longstanding health and socio-economic inequalities between Indigenous and non-Indigenous people are likely to worsen because of the pandemic (Priest, et al. 2020; Markham and Smith 2020). Quite differently, a Brazilian study found no difference in higher risk of COVID-19 based on the presence of risk factors between white and non-white Brazilians (Rezende, et al. 2020).

The pandemic’s exposure of disparities compels us to centre and address systemic issues such as access to healthcare, education, and economic opportunities. Scholars repeatedly highlight key areas critical for addressing these issues such as the necessity to account for the needs and perspectives of minority social groups in planning, funding, and implementation; genuine commitment to respond to the evidence; and willingness to engage approaches that focus on systems change (see Came and Griffiths 2018; Hassen, et al. 2021).

## Trajectories Over Time

The trajectories in the prevalence and forms of racism have changed throughout the pandemic. The pandemic broke out in a climate of mounting xenophobic sentiments, which contributed to the sharp rise in COVID-racism, with numerous reports suggesting that racism spiked in the first couple of months (e.g., Shi, et al. 2022). Early manifestations of racism included scapegoating in media reporting, anti-immigrant statements by politicians, and everyday racist attacks in public places (Ben and Elias, under review; Li and Chen 2021). COVID-racism varied during the second and third waves, declining in certain areas because of travel restrictions and limited social interactions (Gover, et al. 2020). Several factors, including biopolitical measures, restrictions on social interaction, contributed to this, while online activity had the opposite effect (Huang, et al. 2023).

Moreover, racism over time was directed towards additional groups, expanding from a preoccupation with people of Southeast Asian appearance, to target suspected minorities. Time-series trends often varied across ethnic groups (Ballantyne and Giarrusso 2023). In Australia, for example, racism reporting during the pandemic among Chinese Australians and East/Southeast Asian Australians increased compared with pre-pandemic levels. However, it decreased among Asian Australians from other regions, the general population, people born in Australia, and people from English-speaking backgrounds (Ballantyne and Giarrusso 2023; Kamp, et al. 2022; Markus 2020). Limited social interaction during the pandemic, whether due to pandemic restrictions or avoidance of contact because of fears of discrimination, probably reduced racism reporting as well (e.g., Ben and Elias, under review).

The long-term consequences and health effects of pandemic racism receive growing attention too. One study reported the persistence of anti-Asian racism in a longer time span of eighteen months (McGarity-Palmer, et al. 2023), while another found implications after two years for worsening mental health outcomes (Liu, et al. 2023). Multi-ethnic studies indicate that increases in anxiety and depressive symptoms have worsened overtime among those reporting exposures to COVID-19-related racism and that these trajectories varied by gender, with women impacted more negatively (Liu, et al. 2023). In addition, significant racial disparities have been observed over time in health outcomes associated with COVID-19, with racial/ethnic minority groups in the United States and other countries reporting elevated levels of psychological distress (Breslau, et al. 2023).

## Discussion

The impact of COVID-19 on humanity has had disproportionate consequences for minorities (Masters, et al. 2021). From its onset, the pandemic has led to racial disparities in health and socioeconomic outcomes globally and has given rise to novel forms of racism, manifesting interpersonally and structurally (Fisher et al., 2021; Gover, et al. 2020; Gravlee 2020). More than three years post-outbreak, and as the pandemic subsides, we have highlighted three key lessons that can be drawn about pandemic racism during COVID-19.

First, and determined by the pandemic, COVID-racism can be characterized, as fundamentally global; feeding on climates of exclusionary nationalism; and enabled by new media and online technologies that have spread racism worldwide with unprecedented speed and reach. COVID-racism has demonstrated how new contexts are rapidly remoulded into old frames of prejudice, while race and ethnicity are reinscribed. Racism during the pandemic has been further structured through biopolitical measures like policing and surveillance, used disproportionately against minorities. Often expressed blatantly, COVID-racism has signalled that capacities for racist violence, abuse, and hatred remain alive and well, contra to other suggestions.

Second, as the pandemic began spreading, racial disparities have become apparent in infections, severe complications, and mortality from COVID-19. Marginalized groups were disproportionally impacted, which deepened existing inequalities hindering timely and equitable access to resources and needed institutional support (Mackey, et al. 2021; Sabatello, et al. 2021). Across countries, systemic and structural inequities, and the marginalization of ethno-racial minorities had impact in shaping health behaviour, risk for disease, and access to health services (Huang, et al. 2023; Mackey, et al. 2021; Thakur, et al. 2020). The pandemic also revealed the entrenchment of structural racism and the worsening of health outcomes due to systemic inequity.

Third, the trajectories in the prevalence of racism have fluctuated over time, with different manifestations depending on the minority groups targeted. The forms of racism shifted over time, while expressed towards additional groups. Between groups, the prevalence of racism has seen diverse fluctuations, depending for example on the group targeted and social restrictions at a given point in time. Over time, we are starting to unravel the pandemic’s longer-term effects from racism, with evidence for its adverse consequences in areas such as mental health.

Finally, the lessons we have discussed here are significant in the context of post-pandemic recovery, handling future crises, and countering racism. COVID-19 has demonstrated that human cooperation and solidarity are vital to tackling global crises across borders. An equitable and humanizing approach is needed which prioritizes well-being across all differences that can engage with complex systems of oppressions. The pandemic should be taken as a wake-up call to confront and address the deep-rooted structural issues of racism and inequity.

## Footnotes

1 For example, a June 2020 Pew Research Centre poll indicated that 31 per cent of Asian Americans, 21 per cent of African Americans, and 15 per cent of Hispanics experienced COVID-related racial discrimination (Ruiz, et al. 2020).

2 According to the WHO (2023), since March 6, 2023, weekly global infections of COVID-19 has dropped below one million; as of July 10, 2023, weekly cases have dropped to 163,796.

3 Additional dimensions of racism related to the COVID-19 pandemic include bias in medial Artificial Intelligence, algorithmic inequality, digital-divide during lockdowns, unfair travel bans, and detentions of humanitarian migrants, and so on.
